# Increasing the accessibility to internet-based cognitive behavioural therapy for depression: A single-blind randomized controlled trial of condensed versus full-text versions

**DOI:** 10.1016/j.invent.2023.100678

**Published:** 2023-10-04

**Authors:** Magnus Karlsson-Good, Viktor Kaldo, Linnea Lundberg, Martin Kraepelien, Susanne A. Anthony, Fredrik Holländare

**Affiliations:** a Department of Psychiatry, Faculty of Medicine and Health, Örebro University, Örebro, Sweden; b Centre for Psychiatry Research, Department of Clinical Neuroscience, Karolinska Institutet, Stockholm Health Care Services, Region Stockholm, Stockholm, Sweden; c Department of Psychology, Faculty of Health and Life Sciences, Linnaeus University, Växjö, Sweden; d Region Örebro County, Örebro, Sweden; e Division of Psychology, Department of Clinical Neuroscience, Karolinska Institutet, Sweden; f Department of Pediatrics, Skåne University Hospital, Region Skåne, Malmö, Sweden

**Keywords:** Cognitive behavioural therapy, Depressive disorder, Reading speed, Digital intervention, Internet-based treatment, Randomized controlled trial

## Abstract

**Background:**

Research shows that internet-based cognitive behavioural therapy (iCBT) is an effective treatment for depression. However, little is known about how the length of the text material in iCBT affects outcomes.

**Objective:**

The aim of this study was to test whether a condensed iCBT version for depression would be non-inferior to the existing full-text version in reducing depressive symptoms at post-treatment. We also wanted to test non-inferiority for secondary outcomes and explore reading speed and ADHD symptoms as potential moderators.

**Method:**

A single-blind randomized controlled trial was conducted (N = 267) comparing two versions of guided iCBT for depression; full-text (around 60,000 words) and condensed (around 30,000 words, with the option to listen to the text). Estimated between-group effect sizes and their confidence intervals for depression, anxiety and quality of life, were compared to a pre-determined non-inferiority margin (ES = 0.4). Moderation analyses of reading speed and ADHD symptoms were conducted.

**Results:**

The condensed version of iCBT was non-inferior to the full-text version on post-treatment measures for depressive symptoms (95 % CI = −0.42–0.24), anxiety symptoms (95 % CI = −0.24–0.32), and quality of life (95 % CI = −0.09–0.49). Non-inferiority was inconclusive for depressive symptoms at the one-year follow-up (95 % CI = −0.60–0.47). There was no significant moderation effects of reading speed (p = 0.06) or ADHD symptoms (p = 0.11) on depressive symptoms.

**Conclusion:**

These results indicate that a condensed version of iCBT for depression is as effective at treating depression as the full-text version. By shortening texts, iCBT may be made available to more people. Due to unequal dropout rates between the groups, these results are preliminary and need to be replicated.

## Highlights

• There is a lack of research on the effect of text length in internet based cognitive behavioural therapy (iCBT).
• We compared two versions of iCBT for depression with different text length.
• Results showed non-inferior outcomes for the condensed iCBT version.
• Reading speed and ADHD symptoms influence treatment outcome in the condensed iCBT version.

## 1 Introduction

Depression is a common mental disorder (*Diagnostic and Statistical Manual of Mental Disorders*, 2013) affecting about 280 million people worldwide (World Health Organization, 2022). It causes substantial loss in quality of life for the individual, and is a leading cause of disability, amounting to high economic costs at the societal level (James et al., 2018). Cognitive behavioural therapy (CBT) is an efficacious and effective treatment for depression (Cuijpers et al., 2023). CBT delivered through the internet (iCBT) has evolved as a complement to traditional face-to-face therapy (Andersson et al., 2019) and has in several studies shown comparable outcomes (Andersson et al., 2016). Both guided and unguided iCBT for depression have been associated with greater symptom reduction compared to treatment as usual and waiting list control groups (Karyotaki et al., 2021), and there are emerging examples of successful clinics offering iCBT of high quality (Titov et al., 2018). Due to the high demand for CBT and low availability of trained therapists, iCBT has been suggested as a way to spread evidence-based therapy to more patients (Andersson and Titov, 2014). However, questions remain concerning how best to deliver iCBT to patients.

One such question concerns the amount of text material presented to the patient, as there is a risk of overloading the patient with too much information (Andersson and Titov, 2014). Since iCBT is delivered mainly through written modules, the patient is expected to read considerable amounts of text explaining the treatment rationale and CBT strategies. The amount of text in iCBT varies, but can be equivalent to upwards of 150 pages (Andersson et al., 2019). The importance of text being well written and comprehensive but not too long has been highlighted in text-based self-help research over two decades (Andersson et al., 2008a; Martinez et al., 2008; Williams, 2001; Suler, 2001). Too short a text could potentially make the treatment harder to understand. This could lead to deleterious consequences such as reduced treatment satisfaction, adherence and knowledge acquisition (Berg et al., 2019). It might also mean that the therapist spends more time on each patient, for example by having to send and receive more messages in the treatment platform. Despite the importance of these considerations, we do not know of any clear guidelines on text length in iCBT.

Qualitative research and clinical experience reveal varying opinions from patients on the length and difficulty of the text in iCBT treatments (Hadjistavropoulos et al., 2018; Rozental et al., 2015; Patel et al., 2020; McCashin et al., 2019). However, there are examples of studies showing that too much text is a key reason for dropping out (Bendig et al., 2021a; Bendig et al., 2021b). Furthermore, since depression commonly includes fatigue (Demyttenaere et al., 2005), difficulty thinking or concentrating (Rock et al., 2014) and psychosocial difficulties (Cross et al., 2022), there is good reason to consider the effort demanded of the patient in terms of reading. If less time could be spent reading the treatment material with the same treatment effect, that would be an improvement for the patient.

Beyond the cognitive symptoms of depression, there is also a baseline variability among patients in cognitive factors such as reading speed (Andrews, 2012) and aptitude for organization, structure and concentration (ADHD symptoms) (Larsson et al., 2012). Patients in iCBT are required to read, plan and structure the therapy mostly on their own; those with difficulties in these areas could be at a disadvantage and might be helped if the therapy required less reading. However, to the best of our knowledge, no study has examined how the amount of text in iCBT affects treatment outcome.

With this gap in the research literature in mind, this study compares two iCBT versions for depression: one version (full-text) is a previously empirically tested iCBT treatment; the other version is a condensed version of the full-text treatment that contains half the amount of words, as well as the option to listen to the text. The primary aim of this study is to examine whether the condensed version is non-inferior to the full-text version in reducing depressive symptoms, as well as anxiety symptoms and quality of life. Furthermore, we want to investigate whether the influence of ADHD symptoms and reading speed differ between the two iCBT versions. We also want to explore if the difference in text length influences how the participants adhere to the treatment, how much knowledge they gain and their treatment satisfaction. Finally, we are also interested to see if patients or therapists spend more time on the treatment depending on the text length, as well as if it influences the number of messages sent to and from the therapist.

## 2 Method

This was a single-blind randomized trial with parallel arms design, including moderation analyses. Participants were randomized to one of two groups: receiving the full-text or condensed treatment version. The study was approved by the Regional Ethic Review Board in Uppsala, Sweden (2012/406), and registered at clinicaltrials.gov (NCT01788657). This study followed the Consolidated Standards of Reporting Trials (CONSORT) reporting guideline for non-inferiority trials (Piaggio et al., 2012).

### 2.1 Participants

Inclusion criteria for this study were: being an adult (aged 18 years or older) with a diagnosis of mild to moderate major depressive disorder based on the DSM-IV (*Diagnostic and Statistical Manual of Mental Disorders*, 2000); internet access; ability to read and understand Swedish; being a registered inhabitant of Örebro County, Sweden. The exclusion criteria were: severe depressive symptoms (above 34 points on the Montgomery-Åsberg Depression Rating Scale – Self-rating version; MADRS-S) (Svanborg and Åsberg, 1994); high risk of suicide; a diagnosis of bipolar disorder, psychotic disorder, alcohol or drug use disorder, or another psychiatric or somatic disorder that was judged to be contraindicative for iCBT. Participants were also excluded if they were currently undergoing, or planning to undergo, any form of CBT.

Recruitment was conducted by advertising the study to personnel within public healthcare in Örebro County, as well as to local citizens through official websites and Google AdWords. Örebro County is a Swedish regional county servicing about 300,000 inhabitants. It contains a mix of rural and urban settings, and its biggest city Örebro has approximately 150,000 inhabitants.

### 2.2 iCBT versions

The full-text iCBT version consisted of an iCBT treatment for depression used in regular care at the internet psychiatry clinic in Region Stockholm, Sweden (Hedman et al., 2014; Andersson et al., 2005; Vernmark et al., 2010). It contained 59,613 words. The condensed version was created for this study and included the same themes and homework assignments as the full-text version, but the text material was condensed to 30,282 words. There was also an option in the condensed version of listening to the material via supplemental audio files. The condensed treatment was tested in a pilot group of ten patients before the start of the study.

Both the condensed and full-text versions were guided by a therapist and consisted of 10 modules in fixed order. The themes of the ten modules are listed in Supplementary Table S11. The total treatment time was 12 weeks, and participants were recommended to complete one module each week. However, no time restrictions were enforced on the participants for any specific module. The participants received a reminder if they had not logged in to the iCBT platform in seven days.

After a participant completed a module, their therapist would give them written feedback on their homework assignment before opening the next module. Participants were also free to write to their therapist in the platform at any time. Therapists were instructed to answer patients within one workday. The therapists were a licensed clinical psychologist, a therapist with a master of science degree in psychology doing a supervised internship, and a therapist with a bachelor of science degree in social work who had completed a basic psychotherapy course in CBT.

### 2.3 Procedure

Recruitment for the study commenced at 2013-10-29 and the last follow-up data was collected 2017-12-03. Study applicants applied for the treatment through a secure internet-based platform, where they filled out a consent form as well as screening questionnaires. Applicants who did not meet the exclusion criteria were called for an assessment interview. Provided the applicants satisfied the inclusion criteria, they were randomized to the condensed or full-text iCBT, which was started immediately. The participants were blind to which condition they were randomized to. A statistician not otherwise involved in the research process prepared the randomization sequence (1:1) in randomly sized blocks (block sizes ranging between 2 and 50) using a statistical software. This sequence was then used to create cards put in sealed envelopes by an administrator, and opened after the participant was included in the study by the assessor.

During treatment, participants rated their depressive symptoms on MADRS-S (Svanborg and Åsberg, 1994) in the iCBT platform every week. If a participant scored 4 or higher on item 9 on MADRS-S (which measures suicidal ideation on a scale of 0 to 6) a telephone call was made to the participant for an assessment of suicidal ideation and possible referral. At first login (Pre), at the end of the 12-week treatment (Post) and at 12 months after the end of treatment (Follow-up), participants were prompted to fill out questionnaires regarding depressive symptoms, anxiety symptoms, and quality of life. At post-treatment and follow-up, participants also filled out a knowledge test about CBT for depression. A diagnostic interview at the end of treatment and follow-up was planned, but due to lack of resources was not conducted.

### 2.4 Measures

#### 2.4.1 Screening measures

Screening for alcohol and drug abuse was conducted using the Alcohol Use Disorders Identification Test (AUDIT) (Babor et al., 2001) and Drug Use Disorders Identification Test (DUDIT) (Berman et al., 2003). Both AUDIT and DUDIT have shown good psychometric properties in Swedish samples (Berman et al., 2005; Lundin et al., 2015). The Mini-International Neuropsychiatric Interview version 6.0 (Sheehan et al., 1998) was used to help make valid and reliable diagnoses at the assessment interview. AUDIT and DUDIT showed acceptable and good reliability (α = 0.77 and 0.85 respectively) in this sample.

#### 2.4.2 Moderators

Reading speed was measured using a test from “Diagnostiska Läs- och Skrivprov” (Diagnostic manual for analysis of reading and writing skills, DLS) (Järpsten and Taube, 2002). The DLS is a Swedish diagnostic screening tool created to measure the reading comprehension and writing proficiency of primary and secondary school students. It was used in an adult sample due to a lack of valid and reliable alternatives. One of the four tests in the DLS measures reading speed and was used in this study. The participant reads a text from which 36 words are omitted, and the participant has to fill in each gap by choosing one out of three alternative words. The test has a time limit of 3 min and a score range of 0–36. The test was completed using pencil and paper after the diagnostic interview.

The participant's abilities for organization, structure and attentional control were measured using the six-item version of the Adult ADHD Self Report Scale (ASRS-v1.1) (Kessler et al., 2005). ASRS-v1.1 is a screening tool used to identify people with symptoms of ADHD. It has shown good psychometric properties (Kessler et al., 2007). It was dichotomized based on the established cut-off (Kessler et al., 2005), i.e. four or more checkmarks in the darkly shaded area meaning above cut-off (coded 1), and 3 or less being under cut-off (coded 0). It showed questionable (α = 0.69) reliability in this sample.

#### 2.4.3 Primary outcome measure

To measure depressive symptoms MADRS-S was used (Svanborg and Åsberg, 1994). It measures 9 depressive symptoms on a seven-point Likert scale (from 0 to 6), with a score range of 0–54 points. The scale has shown good validity, reliability and sensitivity to change (Svanborg and Åsberg, 2001; Fantino and Moore, 2009). It showed acceptable to excellent reliability (α = 0.71–0.92) in this sample.

#### 2.4.4 Secondary outcome measures

The Beck Anxiety Inventory was used to measure anxiety symptoms (Beck et al., 1988). It contains 21 items on a four-point scale (from 0 to 3), giving a score range of 0–63 points. A recent meta-analysis indicates that the scale has good psychometric properties (Bardhoshi et al., 2016). It showed good to excellent reliability (α = 0.88, 0.91 and 0.92) in this sample.

To measure quality of life EQ-5D-3L was used (EuroQol Group, 1990). It is a widely used health status instrument that contains five items measuring mobility, self-care, usual activities, pain/discomfort, and anxiety/depression on a three-point scale; as well as a visual analogue scale (VAS) measure of general health. It has successfully been applied in a mental health context (Lamers et al., 2006). However, it showed unacceptable to acceptable reliability (α = 0.39, 0.59 and 0.70) in this sample.

Treatment satisfaction was measured using the eight-item version of the Client Satisfaction Questionnaire (CSQ-8) (Larsen et al., 1979), with each item having four levels (from 1 to 4) leading to a score range of 8–32 points. CSQ-8 has shown good psychometric properties (Attkisson and Zwick, 1982). It showed excellent reliability (α = 0.94) in this sample.

A knowledge test was constructed specifically for this study. Participants were asked to evaluate 20 statements as either correct or incorrect. The statements were chosen based on the content of the iCBT modules in our treatment versions as well as expert opinion regarding what knowledge was important to learn during a CBT treatment for depression. Wrong answers were coded as 0 and correct answers as 1, giving the test a range of 0–20 points. See Supplementary material Table S15 for the full test. Its reliability in this sample was unfortunately unacceptable and questionable (α = 0.48 and 0.63).

#### 2.4.5 Adherence measures

Adherence was operationalized as the number of modules completed during the treatment and how much homework was completed. The number of completed treatment modules was recorded by the treatment platform. Participants were asked weekly whether they had completed any homework assignments during the last week. These choices were coded 0–2, with 0 meaning having done no homework assignments, 1 meaning having done some homework and 2 meaning having done all homework assignments.

#### 2.4.6 Time measures

The time participants spent on the iCBT treatment was measured in two ways. Firstly, when participants logged in to the iCBT platform used for delivering the treatment a timer automatically started, which stopped when the participant logged out. The timer was not sensitive to when the participant was actively interacting with the iCBT modules. Secondly, participants were prompted weekly to estimate the total number of minutes spent on all aspects of the treatment during that week. The time therapists spent on each patient was measured by the system in the same way as for patients.

#### 2.4.7 Messages

The number of messages sent to the therapist and participants were automatically recorded by the platform.

### 2.5 Data analysis

#### 2.5.1 Choice of non-inferiority margins and sample size

A non-inferiority margin of an effect size of 0.4 was chosen in this study based on consensus among the authors, that it represents a small effect according to Cohen's thresholds (Cohen, 1988), and earlier research (Hedman et al., 2011).

In an a priori power calculation using NQuery advisor (Elashoff, 2000), we set the alpha level at 0.05, and estimated the standard deviation for MADRS-S at 8 points (Andersson et al., 2005; Vernmark et al., 2010). To get 80 % power to detect a difference of d = 0.4, we needed 78 participants in each study arm. We estimated an attrition of at least 20 %, and therefore obtained ethical approval to include between 195 and 300 participants.

#### 2.5.2 Statistical analyses

To analyse change in depressive symptoms, anxiety symptoms, and quality of life we used linear mixed-effects regression models, as they can handle dependence of data due to repeated measures over time, use all available data and handles missing data under fairly unrestrictive assumptions (Hesser, 2015). All randomized participants who contributed at least one measurement were entered into the models, i.e. intention-to-treat analysis. Assumptions for the models were met.

The common fixed effects of all models were time, time squared, and treatment group. Time squared was a squared version of the regular time variable. It was added to account for the non-linear trend in the slopes due to the two distinct phases of the treatment (pre to post-treatment and post-treatment to follow-up).

We started with a simple unconditional regression model and then added all the fixed effects. Random effects were added one by one to the model and kept if they significantly improved the fit of the model. Effect sizes and their respective confidence intervals were calculated using the model-estimated mean difference at post-treatment and follow-up.

The moderation analysis used the same principles as the main outcome analyses. However, only pre to post-treatment data were used. Moderation was analysed by estimating a time × group × moderator variable.

*t*-Tests were conducted to test differences between the groups on time spent on the iCBT treatment, number of modules assigned, treatment satisfaction, results on the knowledge tests, and messages sent and received. To test for differences in self-reported completion of homework assignments, chi-square analyses were conducted for each of the treatment weeks.

See Supplementary material (Section 1) for more information.

## 3 Results

**Fig. 1:**
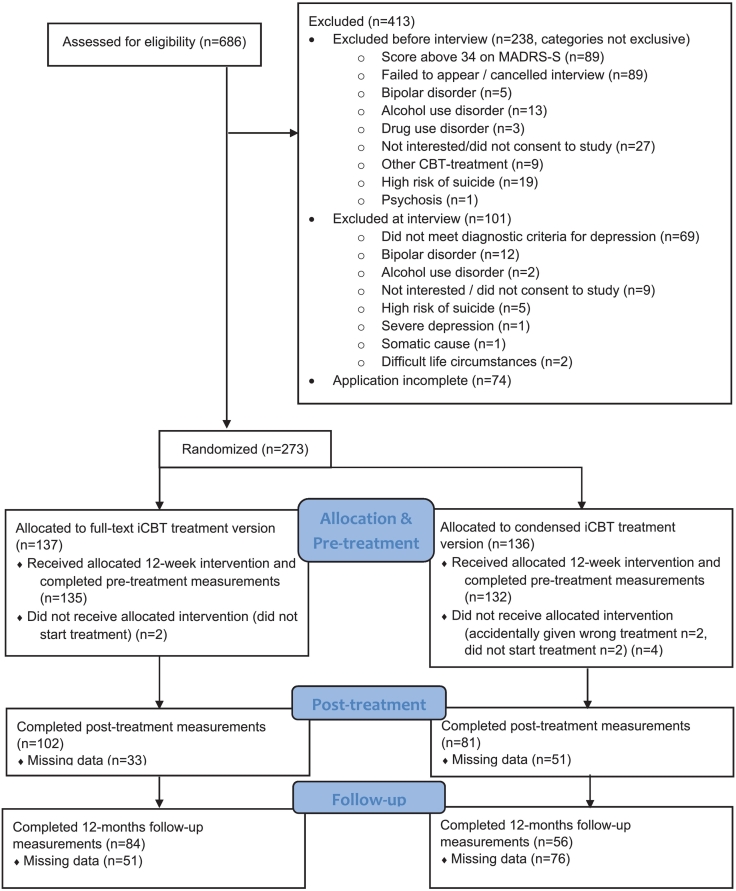
Study flow chart. (MADRS-S = Montgomery-Åsberg Depression Rating Scale – Self-rating version.)

See Fig. 1 for the participant flowchart of the study. A total number of 663 participants were assessed for eligibility, of which 273 were randomized to the two conditions. Four participants never logged in to the treatment platform and therefore did not provide baseline data, two were mistakenly put in the wrong treatment group. Therefore we excluded these six participants from analysis. This meant 267 participants were eligible for statistical analysis for the primary outcome, 135 in the full-text group and 132 in the condensed group. For secondary outcomes, three participants were excluded from analysis of anxiety symptoms due to missing data at all three time points. One participant was excluded from the analysis of quality of life for the same reason. Participants were predominantly female with a secondary or post-secondary education, a mean age of 38 years and at least one comorbid mental disorder. Most participants were not taking any medications for their mental health. See Table 1 for participant characteristics. There were no noticeable group differences on demographic or clinical characteristics. There was a significant difference in dropout at post-treatment and follow-up, in that more participants in the condensed group did not fill these out (see Section 9 in Supplementary material).

**Table 1:** Participant characteristics.

Measure	Total (n = 267)	FULL (n = 135)	COND (n = 132)
Age, mean (SD)	38.2 (12.4)	37.4 (10.7)	39.3 (14.1)
Female, %	74.5	74.1	75.0
Education level, n (%)			
Primary	16 (6.0 %)	10 (7.4 %)	6 (4.6 %)
Secondary	89 (33.5 %)	44 (32.6 %)	45 (34.4 %)
Vocational education	39 (14.7 %)	21 (15.6 %)	18 (13.7 %)
Post-secondary	111 (41.7 %)	55 (40.7 %)	56 (42.7 %)
Other	11 (4.1 %)	5 (3.7 %)	6 (4.6 %)
Form of household, n (%)			
Living alone	99 (37.2 %)	50 (37.0 %)	49 (37.4 %)
Cohabitating	167 (62.8 %)	85 (63.0 %)	82 (62.6 %)
Comorbidity, n (%)			
GAD	115 (43.1 %)	57 (42.2 %)	58 (43.9 %)
PD	50 (18.7 %)	26 (19.3 %)	24 (18.2 %)
Agoraphobia	33 (12.4 %)	15 (11.1 %)	18 (13.6 %)
Social anxiety	66 (24.7 %)	32 (23.7 %)	34 (25.8 %)
PTSD	21 (7.9 %)	12 (8.9 %)	9 (6.8 %)
Other	33 (12.4 %)	20 (14.8 %)	13 (9.8 %)
Any comorbidity	168 (62.9 %)	88 (65.2 %)	81 (61.4 %)
No comorbidity	99 (37.1 %)	47 (34.8 %)	51 (38.6 %)
Current medication, n (%)			
Antidepressant	97 (36.3 %)	47 (34.8 %)	50 (37.9 %)
Anxiolytic	16 (6.0 %)	8 (6.0 %)	8 (6.1 %)
Other	5 (1.9 %)	3 (2.2 %)	2 (1.5 %)
Any medication	104 (39.0 %)	51 (37.8 %)	53 (40.2 %)
No medication	163 (61.0 %)	84 (62.2 %)	79 (59.8 %)
Screening measures, M (SD)			
AUDIT	4.44 (4.12)	4.25 (4.06)	4.63 (4.18)
DUDIT	0.47 (2.18)	0.24 (1.22)	0.72 (2.83)
Moderators, M (SD)			
DLS	24.46 (6.03)	24.54 (5.96)	24.38 (6.12)
ASRS	12.87 (4.14)	12.65 (4.34)	13.09 (3.92)

FULL = full-text iCBT treatment version. COND = condensed iCBT treatment version. GAD = generalized anxiety disorder. PD = panic disorder. PTSD = post-traumatic stress disorder. AUDIT = Alcohol Use Disorders Identification Test. DUDIT = Drug Use Disorders Identification Test. DLS = *Diagnostiska Läs- och Skrivprov* (Diagnostic manual for analysis of reading and writing skills). ASRS = Adult ADHD Self Report Scale.

### 3.1 Primary outcome

**Fig. 2:**
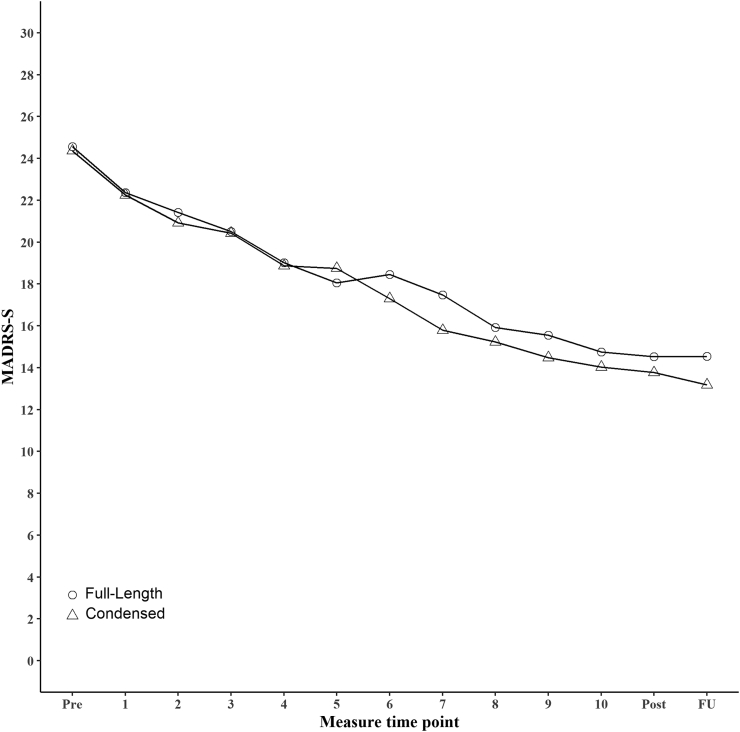
Observed means for self-rated depressive symptoms (MADRS-S) from pre-treatment to follow-up for the two iCBT treatment versions. Note. Circles represent the group receiving the full-text treatment version; triangles represent the group given the condensed version. Measurements were taken weekly during the treatment. (MADRS-S = Montgomery-Åsberg Depression Rating Scale – Self-rating version. Pre = pre-treatment. Post = post-treatment (week 12). FU = One-year follow-up.)

Observed means and estimated between group effect sizes of the primary outcome, MADRS-S, are presented in Table 2. Within group effect sizes for the full-text group and condensed group at endpoint was ES = 1.33, 95 % CI = 0.96–1.71 and ES = 1.43, 95 % CI = 1.05–1.81 respectively. The linear and quadratic terms in the linear mixed-effects model that tested differential change between the full-text and condensed iCBT treatment groups over time were not statistically significant, β = −0.05, p = 0.62 and β = 0.00, p = 0.62 respectively. The upper limit of the confidence interval of the effect sizes at post-treatment was below 0.4, indicating that the condensed iCBT version is non-inferior to the full-text version, ES = −0.09, 95 % CI = −0.42–0.24. This also held true for the sensitivity analysis without modules assigned as a co-variate in the model (see Table S14 in the Supplementary material). At follow-up, the confidence interval included 0.4, and non-inferiority could not be established, ES = −0.06, 95 % CI = −0.60–0.47. See Fig. 2 for weekly observed mean scores of MADRS-S for the groups. See also the Supplementary material (Sections 2, 5 and 10) for full model outputs, within-group effect sizes and non-inferiority figs.

**Table 2:** Observed means and estimated effect sizes with 95 % confidence intervals for the two iCBT treatment versions.

Time	Full-text iCBT treatment version		Condensed iCBT treatment version		Effect size (95 % CI)
	N	Mean (SD)	N	Mean (SD)	
*MADRS-S*					
Pre	135	24.56 (5.36)	132	24.36 (5.58)	
Post	102	14.52 (8.21)	81	13.77 (7.71)	−0.09 (−0.42–0.24)
Follow-up	84	14.54 (9.75)	56	13.18 (8.41)	−0.06 (−0.60–0.47)

*BAI*					
Pre	134	19.21 (8.28)	130	19.42 (8.82)	
Post	98	14.53 (7.92)	76	15.64 (8.91)	0.04 (−0.24–0.32)
Follow-up	82	14.77 (9.32)	55	13.33 (9.23)	−0.21 (−0.55–0.14)

*EQ-5D*					
Pre	135	0.55 (0.27)	131	0.56 (0.25)	
Post	101	0.64 (0.29)	77	0.69 (0.25)	0.20 (−0.09–0.49)
Follow-up	87	0.69 (0.30)	55	0.71 (0.26)	0.15 (−0.17–0.47)

*EQ-5D VAS*					
Pre	135	46.66 (18.17)	131	45.37 (16.58)	
Post	101	62.36 (19.03)	77	62.19 (17.21)	0.03 (−0.27–0.34)
Follow-up	87	64.45 (21.01)	55	64.55 (19.28)	0.06 (−0.28–0.39)

Note. Means are based on observed scores from pre-treatment, post-treatment and one-year follow-up. Effect sizes were computed using model-estimated means at post-treatment and follow-up divided by pre-treatment observed standard deviation. A negative effect size for MADRS-S and BAI indicates that the condensed version was superior, while a negative effect size for EQ-5D and EQ-5D VAS indicates that the full-text version was superior. iCBT = Internet-based cognitive behavioural therapy. MADRS-S = Montgomery-Åsberg Depression Rating Scale – Self-rating version. BAI = Beck Anxiety Inventory. VAS = Visual Analogue Scale.

### 3.2 Secondary outcomes

Observed means and estimated effect sizes of secondary outcomes are presented in Table 2. The terms in the linear mixed-effects model that tested differential change between the full-text and condensed iCBT treatment groups over time were not statistically significant for any measure. The upper limit of the confidence interval was below 0.4 on the Beck Anxiety Inventory for both post-treatment and follow-up, ES = 0.04, 95 % CI = −0.24–0.32 and −0.21, 95 % CI = −0.55–0.14 respectively.

For EQ-5D and EQ-5D VAS the lower limit of the confidence interval was above −0.4 at both post-treatment, ES = 0.20, 95 % CI = −0.09–0.49 and ES = 0.03, 95 % CI = −0.27–0.34 respectively, and follow-up, ES = 0.15, 95 % CI = −0.17–0.47 and ES = 0.06, 95 % CI = −0.28–0.39 respectively. This indicates that the condensed iCBT version was non-inferior on all secondary outcomes both at post-treatment and follow-up. See also the Supplementary material (Sections 2 and 10) for full model outputs and non-inferiority figs.

### 3.3 Moderation analyses

The terms in the linear mixed-effects models that tested for the interaction of time and reading speed (DLS score) or ADHD symptoms (ASRS score), regardless of treatment group, on outcome were not significant. See Supplementary material (Tables S5 and S6) for full model outputs. The three-way interaction between treatment group, time, and reading speed was not significant, F(1, 206.681) = 3.47, p = 0.06. There was also no significant interaction between treatment group, time, and ASRS score, F(1, 205.541) = 2.61, p = 0.11. See the Supplementary material (Sections 7 and 8) as well as table S10 and S11 for sensitivity analyses.

Post-hoc analyses showed significant two-way interactions between time and the moderators in the condensed iCBT version; for reading speed, F(1, 96.297) = 4.29, p = 0.041, for ASRS score, F(1, 98.183) = 4.11, p = 0.045. The same two-way interactions were not significant for the full-text version. See Section 11 in the Supplementary material for full model outputs.

**Fig. 3:**
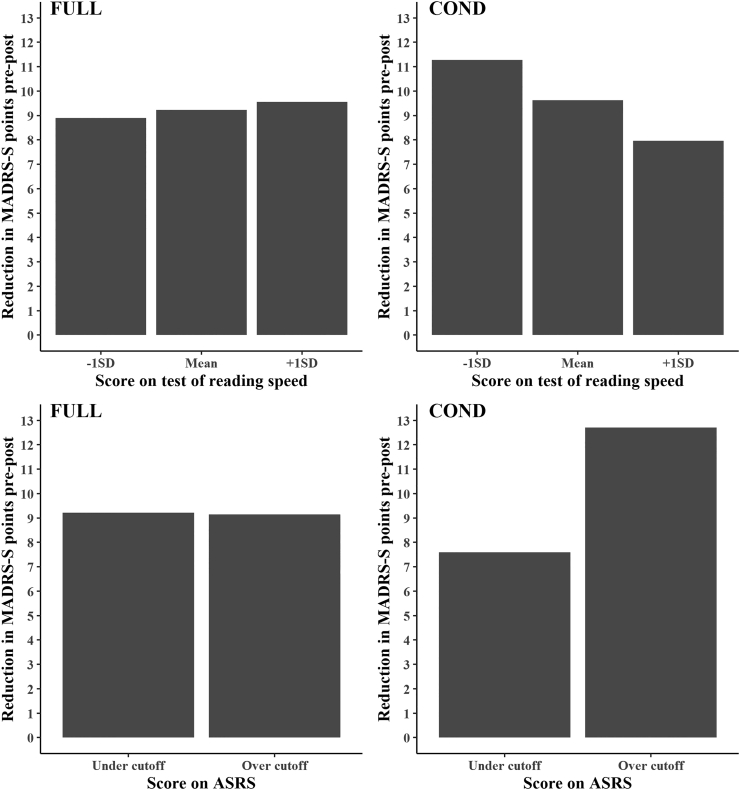
Estimated reduction in self-rated depressive symptoms (MADRS-S) from pre to post treatment for different levels of reading speed and ADHD symptoms in full-text iCBT treatment version and condensed treatment version. Note. DLS is a Swedish diagnostic instrument for the analysis of reading and writing skills. −1SD = DLS reading speed score of one standard deviation below the mean. +1SD = DLS score one standard deviation above the mean. (FULL = Full-text iCBT version. COND = Condensed iCBT version. MADRS-S = Montgomery-Åsberg Depression Rating Scale – Self-rating version. DLS = *Diagnostiska Läs- och Skrivprov*. ASRS = Adult ADHD Self-Report Scale.)

To visualize these moderations, we calculated treatment outcomes for different levels of the moderators in the two iCBT versions and show these graphically; see Fig. 3. Greater difficulties (lower reading speed and higher ASRS score) indicate greater reductions in depressive symptoms in the condensed version, but not in the full-length version. A participant with one standard deviation below the average DLS score in the condensed ICBT version had an estimated pre to post-treatment effect size of 2.12. For a participant with a score one standard deviation above the average the estimated effect size was 1.50. For ASRS, the estimated effect sizes for someone below the cut-off and above the cut-off were 1.42 and 2.39 respectively.

### 3.4 Other measures

*t*-Tests revealed no statistically significant differences between the treatment groups on any measure of time spent on the treatment by the participant or therapist, number of modules assigned, treatment satisfaction, the knowledge test, messages sent or messages received. The results are presented in Table 3.

**Table 3:** Results from t-tests on measures of time spent on the iCBT treatments, number of modules assigned, treatment satisfaction, knowledge and messages sent and received.

	Full-text iCBT treatment version		Condensed iCBT treatment version		T (df)	p
	n	Mean (SD)	n	Mean (SD)		
Time-SR weekly	131	87.87 (91.76)	125	76.75 (84.26)	1.01 (254)	0.31
Time-sys participant	134	1648.77 (2336.74)	132	1270.46 (1846.38)	1.46 (264)	0.14
Time-sys therapist	133	5.05 (4.19)	127	5.35 (3.91)	−0.60 (258)	0.55
Modules assigned	134	6.68 (3.37)	130	6.54 (3.19)	0.33 (262)	0.74
Treatment satisfaction	103	24.45 (4.72)	84	24.69 (4.99)	−0.34 (185)	0.73
Knowledge test post	97	17.74 (1.52)	76	17.39 (2.05)	1.28 (171)	0.20
Knowledge test follow-up	87	17.18 (2.29)	55	17.16 (1.86)	0.06 (140)	0.96
Messages sent	135	12.06 (7.78)	132	11.77 (7.32)	0.32 (265)	0.75
Messages received	135	12.79 (5.65)	132	12.42 (4.99)	0.56 (265)	0.57

iCBT = Internet-based cognitive behavioural therapy. Time-SR weekly = mean of self-reported time spent on the iCBT treatment each week, in minutes. Time-sys participant = total time the participant spent logged into the treatment platform, in minutes. Time-sys therapist = average time the therapist spent logged into the treatment platform for each participant, in minutes.

Chi-squared analyses were conducted to test for differences in weekly self-reported completion of homework assignment. No significant differences were found between the treatment groups, with p-values ranging from 0.14 to 0.82.

## 4 Discussion

The main result of this study is that the condensed version, with added audio files, of iCBT for depression was non-inferior compared to the original full-text version for the primary outcome, depressive symptoms, and for the secondary outcome measures of anxiety and quality of life at the end of the 12-week treatment. At follow-up after one year, non-inferiority was established for all outcomes except depressive symptoms. The higher variability in outcome at follow-up, as well as missing data, led to a wider confidence interval. Still, it is worth noticing that the estimated effect size was in favour of the condensed version. Taken together, it is reasonable to state the condensed version as non-inferior.

There was no statistically significant moderating effect of ADHD symptoms or reading speed on reduction in depressive symptoms. There was however a clear trend, and one possible explanation for this lack of significant effect is that we did not have enough power to detect it. Post-hoc tests showed a significant two-way interaction in the condensed iCBT version. It indicated a better treatment effect in the condensed iCBT version for those participants with greater difficulties (lower reading speed and more ADHD symptoms). This is tentative evidence that subgroups of patients with deficits in reading and organizing their work on an iCBT treatment could benefit more from a condensed version. Future research on these factors and their relationship with treatment outcomes are needed to draw firm conclusions.

We also did not find a statistically significant difference between the treatment groups in time spent on the treatment or the number of assigned modules. The low precision in the time measures we used might conceal the expected relationship that it takes less time to read a shorter text compared to a longer. The time logged in the platform do not account for how active the patient is while logged in, and the patient-rated time is both retrospective and includes not only time reading but time spent on all aspects of the treatment.

It is difficult to compare our findings to previous research as the length of the treatment material is rarely reported in internet-based treatments for depression. A scoping review of internet-based treatments for depression found that only 2.7 % (n = 3) of studies provided an extensive description of their treatment material (Børtveit et al., 2022). None of these three studies included a word count. More information on the treatments used in research would help inform future treatment development. To our knowledge, no earlier studies have looked specifically at the effect of text length in iCBT on treatment outcome. Earlier research on the influence of cognitive factors on iCBT shows mixed results (El Alaoui et al., 2015; Silfvernagel et al., 2018; Lindner et al., 2016; Andersson et al., 2008b). Some studies found small effects on adherence and outcome, but others show no effect.

This study has several limitations. The non-inferiority margin was chosen when non-inferiority trials were rare in iCBT research, and the available research had a similar margin (Hedman et al., 2011). A different choice of margin could have produced a different result. One common margin is an effect size of d = 0.24, based on a study of clinical significance (Cuijpers et al., 2014). Our results at the end of treatment for depressive symptoms slightly overlap this margin. Another limitation is the difference in dropout between the two groups. No statistical method can fully compensate for missing data, and caution must therefore be used in interpreting these results. See Sections 3, 5 and 9 in the Supplementary material for additional information. The lack of reliability on our measures of quality of life and knowledge acquisition also needs to be taken into consideration. Lastly, it is not possible to disentangle the effect of condensing the treatment and adding audio files, which limits the internal validity of the study.

Future research should replicate these results and expand them to iCBT for other conditions. There is a lack of well-controlled studies on similar practical design questions regardless of treatment target. For example, research on the number and extent of homework assignments, language complexity, and number of modules is lacking (Børtveit et al., 2022). To increase accessibility to treatment, we need to find a way of designing treatments that contains the necessary information and treatment elements, while removing material that adds to the effort required of the patients without providing clear benefits. It is possible that the text in both treatments of the current trial would be considered long if compared to other iCBT programs, and future studies should focus on comparing to even shorter texts.

In conclusion, this study show that a condensed version of iCBT for depression, with shorter texts and an option to listen to the material as audio files, can have non-inferior results compared to a full-text version. Shortening treatment materials might increase the availability of iCBT.

## Funding

This work was supported by the L.J. Boëthius foundation, Sweden; and ALF funding, 10.13039/501100009228Region Örebro County, Sweden. The funders had no involvement in study design; in the collection, analysis and interpretation of data; in the writing of the report; or in the decision to submit the article for publication.

## Declaration of competing interest

The authors declare the following financial interests/personal relationships which may be considered as potential competing interests: FH licenses a cognitive-behavioural relapse prevention manual for depression to Pear Therapeutics Inc.

## Appendix A Supplementary data

Supplementary material Image 1
