# Supplementary material for: Increasing the accessibility to internet-based cognitive behavioural therapy for depression: A single-blind randomized controlled trial of condensed versus full-text versions
Source: Internet Interv. 2023 Oct 4;34:100678. doi: 10.1016/j.invent.2023.100678 (PMC10570001; doi:10.1016/j.invent.2023.100678)
Supplement: Supplementary file 1 — Supplementary material [file mmc1.docx]

**Online supplement**

**Table of contents**

**1. Data analysis2**

1.1 Choice of non-inferiority margins and sample size2

1.2 Main outcomes2

1.3 Moderation analysis3

1.4 Other measures4

**2. Full model output from mixed-effect models5**

**3. Full model output from mixed-effect models without controlling for assigned modules11**

**4. Description of iCBT treatment modules15**

**5. Observed means for each week, within-group effect sizes and effect sizes without controlling for assigned modules 16**

**6. Knowledge test17**

**7. Sensitivity analyses of moderation of reading speed18**

7.1 Sensitivity analysis of moderating effect of reading speed18

7.2 Reading speed and adherence/engagement18

**8. Sensitivity analyses of moderation of ADHD-symptoms20**

**9. Difference in missing data 20**

**10. Non-inferiority figures21**

**11. Full model output of post-hoc two-way interaction analyses of moderation23**

**References27**

**1. Data analysis**

**1.1 Choice of non-inferiority margins and sample size**

A non-inferiority margin of an effect size of 0.4 was chosen in this study based on consensus among the authors, that it represents a small effect according to Cohen’s thresholds (1), and earlier research (2).

Based on calculations using NQuery advisor (3), given a standard deviation of 8 points on MADRS-S, a power of 0.80 and an alpha level of .05, 78 participants were required in each treatment arm to find a difference of *d* = 0.4. An estimation of 20% attrition was made, therefore setting a goal of a minimum of 195 participants in the study.

The choice of non-inferiority margin and power analysis were made before collecting any data, but these were unfortunately not made available at the preregistration on clinicaltrials.gov. They were, however, available in the research plan that was sent to the Regional Ethical Review Board in Uppsala.

**1.2 Main outcomes**

To analyse changes in depressive symptoms, anxiety symptoms, and quality of life we used linear mixed-effects models, as they can handle dependence of data due to repeated-measures over time (4). All randomized participants who contributed at least one measurement were entered into the models.

We checked assumptions of linearity and homoscedasticity by plotting the predicted values of the final models against the residuals. We also plotted histograms and Q-Q plots of the residuals to check the assumption of normality. Correlations between the variables entered into the model were weak, and therefore multicollinearity was not an issue. The exception were the two time variables which were highly correlated, but in line with Biesanz et al. (5) not considered relevant for the assumption of multicollinearity. All final models met assumptions of linearity, homoscedasticity, normality and multicollinearity.

The common fixed effects of all models were time, time squared, and treatment group. For depressive symptoms, time had 13 measurements and was coded 0–63, with 0–11 being the pre-treatment measurement, the weekly measurements and post-treatment measurements, and the follow-up coded as 63. For anxiety symptoms and quality of life, time had three measurement points and was coded 0, 11, and 63.

Time squared was a squared version of the regular time variable. It was added to account for the non-linear trend in the data due to the two distinct phases of the treatment (pre to post-treatment and post-treatment to follow-up). In all models, the time squared variable also significantly improved the model fit. Treatment group was a dichotomous variable with the full-text iCBT group coded 0 and the condensed iCBT group coded 1.

We started with a simple unconditional model and first added all the fixed effects, then the random effects. Random effects were added one by one to the model and kept if they significantly improved the fit of the model according to the likelihood ratio test. For depressive symptoms and anxiety symptoms, random effects (i.e. intercept and slope) used an unstructured covariance structure. For quality of life, random effects (intercept) used a scaled identity covariance structure. For depressive symptoms individual errors were analysed using an autoregressive covariance structure. For anxiety symptoms and quality of life, a scaled identity covariance structure was used for individual errors. Covariance structures were chosen based on model fit and the models’ ability to converge. All nested models were analysed with full information maximum likelihood and were compared using the -2 restricted log likelihood. However, the final models were analysed with restricted maximum likelihood and Satterthwaite approximations as it gives better estimates of fixed effects (6), which was the focus of our analysis.

Missing data was handled in accordance with recommendations from Karin et al. (7, 8). First, we established which variables that could be assumed to be related to missing data. These were: age, level of education, gender, baseline symptoms of anxiety and depression, comorbidity, total time spent on treatment for the therapist and patient (both self-reported and system-reported), total amount of log-ins to the treatment platform, total amount of messages sent to and received from the therapist, number of modules assigned during treatment, reading speed, ADHD symptoms and alcohol consumption. These variables were then entered into separate logistic regression models with missing data at post-treatment as a dichotomous outcome. After that, a combined logistic regression model was made containing all the variables that were significant in the separate models. There, only number of assigned modules during treatment remained a significant predictor. Assigned modules was therefore entered into a linear mixed effects model to see if it also significantly predicted treatment outcome. The number of assigned modules was significant in this model but only for depressive symptoms and the EQ5D VAS measure of quality of life. The number of assigned modules was therefore added as a fixed effect to the models that used depressive symptoms and the VAS-measure of quality of life, to achieve less biased estimates based on missing data (7, 8). The variable was centred to its median for easier interpretation.

Effect sizes and their respective confidence intervals were calculated using the model-estimated mean difference at post-treatment and follow-up, and its respective confidence intervals, using equations 11 and 13 from Feingold (9), see also equation 1-4 below. This effect size is an adapted form of Cohen’s d appropriate for growth modeling analysis (GMA) and similar models. Standard deviations in the nominator were based on observed data at baseline. The method used is specifically appropriate due the non-linear trend in our data (9).

$GMA d = \frac{b_{i}-isb_{i}}{SD}$ (1)

${Var}_{GMA d}=\frac{{SE}_{b_{i}}^{2}}{{SD}^{2}}$ (2)

${SE}_{GMA d} = \sqrt{{Var}_{GMA d}}$ (3)

${CI}_{GMA d} =GMA d\pm1.96 * {SE}_{GMA d}$ (4)

Note: b_i_ represents the estimated parameter of group effect at post-treatment and follow-up respectively; isb_i_ represents the estimated parameter of group effect at pre-treatment. SD = observed standard deviation at baseline measurement. Var = Variance. SE = Standard error.

**1.3 Moderation analysis**

The moderation analysis used the same principles as the main outcome analyses concerning assumptions, missing data and strategy for building the models. However, only pre to post-treatment data were used. Treatment group, time, moderator, interaction between moderator and time, and interaction between moderator, time, and treatment group were added as fixed effects. Random effects (i.e. intercept and slope) used an unstructured (UN) variance type, while individual errors were analysed using an autoregressive (AR) variance type. Number of modules assigned during treatment was added as a covariate to achieve less biased estimates due to missing data.

A post-hoc two-way interaction (time × moderator) analysis was made for each treatment group using the same specifications as the full moderation analysis.

**1.4 Other measures**

T-tests were conducted to test differences between the groups in time spent on the treatment, number of modules assigned, treatment satisfaction, results on the knowledge test, and messages sent and received. One outlier was removed from the system-measured time spent on treatment because the number was unreasonably high (10 times the second-largest number) and did not match the participants’ self-reported time spent on the treatment. Eleven participants did not report their time spent on the treatment and therefore did not have data for the analysis. Otherwise, all assumptions were met for the analyses. To test for differences in self-reported completion of homework assignment, chi-square analyses were conducted for each of the treatment weeks.

The alpha level for all analyses was set at 0.05. All statistical analyses were made using SPSS version 27 (10). Figures were created using R version 4.0 (11), with the package ggplot2 (12).

**2. Full model output from mixed-effect models**

**Table S1. Full model output for depressive symptoms (MADRS-S)**

| Fixed effects | | | | | |
| --- | --- | --- | --- | --- | --- |
|  | Est/Beta | SE | 95% CI | t | p |
| Intercept | 23.83 | 0.59 | 22.67 – 24.99 | 40.26 | < 0.001 |
| Time | -0.99 | 0.07 | -1.12 – -0.85 | -14.12 | < 0.001 |
| Time sq | 0.01 | 0.00 | 0.01 – 0.02 | 13.52 | < 0.001 |
| Group | -0.06 | 0.84 | -1.72 – 1.59 | -0.07 | 0.94 |
|  |  | | | | |
| Time × group | -.05 | 0.10 | -0.25 – 0.15 | -0.49 | 0.62 |
| Time sq × group | 0.00 | 0.00 | -0.00 – 0.00 | 0.49 | 0.62 |
|  |  | | | | |
| No. of modules assigned | -0.41 | 0.12 | -0.63 – -0.18 | -3.52 | < 0.001 |
|  | | | | | |
| Random effects | | | | | |
|  | | | | Estimate | SE |
| AR(1) Diagonal | | | | 26.84 | 1.77 |
| AR(1) Rho | | | | 0.63 | 0.03 |
| UN (1,1) | | | | 25.91 | 3.34 |
| UN (2,1) | | | | 0.00 | 0.06 |
| UN (2,2) | | | | 0.01 | 0.00 |

(MADRS-S = Montgomery-Åsberg Depression Rating Scale – Self-rating version.)

**Table S2. *Full model output for anxiety symptoms (Beck Anxiety Inventory)***

| Fixed effects | | | | | |
| --- | --- | --- | --- | --- | --- |
|  | Est/Beta | SE | 95% CI | t | p |
| Intercept | 19.21 | 0.74 | 17.75 – 20.67 | 25.95 | < .001 |
| Time | -.43 | 0.08 | -0.59 – -0.27 | -5.43 | < .001 |
| Time sq | 0.01 | 0.00 | 0.00 – 0.01 | 4.87 | < .001 |
| Group | 0.14 | 1.05 | -1.93 – 2.21 | 0.13 | 0.90 |
|  |  | | | | |
| Time × group | 0.05 | 0.12 | -0.19 – 0.28 | 0.38 | 0.70 |
| Time sq × group | -0.00 | 0.00 | -0.00 – 0.00 | -0.65 | 0.52 |
|  | | | | | |
| Random effects | | | | | |
|  | | | | Estimate | SE |
| Residual | | | | 27.63 | 3.04 |
| UN (1,1) | | | | 45.77 | 6.28 |
| UN (2,1) | | | | 0.03 | 0.10 |
| UN (2,2) | | | | 0.00 | 0.00 |

**Table S3. *Full model output for quality of life (EQ-5D)***

| Fixed effects | | | | | |
| --- | --- | --- | --- | --- | --- |
|  | Est/Beta | SE | 95% CI | t | p |
| Intercept | 0.55 | 0.02 | 0.50 – 0.59 | 23.66 | < .001 |
| Time | .01 | 0.00 | 0.00 – 0.01 | 3.29 | < .01 |
| Time sq | -0.00 | 0.00 | -0.00 – 0.00 | -2.61 | 0.01 |
| Group | 0.01 | 0.03 | -0.05 – 0.08 | 0.42 | 0.68 |
|  |  | | | | |
| Time × group | 0.01 | 0.00 | 0.00 – 0.01 | 1.34 | 0.18 |
| Time sq × group | -0.00 | 0.00 | -0.00 – 0.00 | -1.24 | 0.21 |
|  | | | | | |
| Random effects | | | | | |
|  | | | | Estimate | SE |
| Residual | | | | 0.03 | 0.00 |
| Intercept | | | | 0.04 | 0.00 |

**Table S4. *Full model output for quality of life (EQ-5D VAS)***

| Fixed effects | | | | | |
| --- | --- | --- | --- | --- | --- |
|  | Est/Beta | SE | 95% CI | t | p |
| Intercept | 46.68 | 1.58 | 43.57 – 49.80 | 29.48 | < .001 |
| Time | 1.69 | 0.20 | 1.30 – 2.09 | 8.41 | < .001 |
| Time sq | -0.02 | 0.00 | -0.03 – -0.02 | -7.34 | < .001 |
| Group | -1.29 | 2.25 | -5.71 – 3.14 | -0.57 | 0.57 |
|  |  | | | | |
| Time × group | 0.06 | 0.30 | -0.53 – 0.66 | 0.21 | 0.83 |
| Time sq × group | -0.00 | 0.00 | -0.01 – 0.01 | -0.17 | 0.87 |
|  | | | | | |
| No. of modules assigned | 0.08 | 0.31 | -0.53 – 0.69 | 0.26 | 0.79 |
|  | | | | | |
| Random effects | | | | | |
|  | | | | Estimate | SE |
| Residual | | | | 185.65 | 14.49 |
| Intercept | | | | 151.74 | 21.89 |

(VAS = Visual analogue scale.)

**Table S5. *Full model output of moderation analysis of ADHD symptoms***

| Fixed effects | | | | | |
| --- | --- | --- | --- | --- | --- |
|  | Est/Beta | SE | 95% CI | t | p |
| Intercept | 21.62 | 0.56 | 20.51 – 22.72 | 38.57 | < 0.001 |
| Time | -0.84 | 0.09 | -1.02 – -.65 | -8.84 | < 0.001 |
| Group | -0.19 | 0.65 | -1.47 – 1.08 | -0.30 | 0.76 |
| ASRS | 3.83 | 0.65 | 2.55 – 5.11 | 5.89 | < 0.001 |
|  |  | | | | |
| Time × group | 0.15 | 0.14 | -0.14 – 0.43 | 1.02 | 0.31 |
| Time × ASRS | 0.01 | 0.14 | -0.26 – 0.27 | 0.04 | 0.96 |
| Time × group × ASRS | -0.32 | 0.20 | -0.71 – 0.07 | -1.62 | 0.11 |
|  | | | | | |
| No. of modules assigned | -0.22 | 0.10 | -0.41 – -0.02 | -2.18 | 0.03 |
|  | | | | | |
| Random effects | | | | | |
|  | | | | Estimate | SE |
| AR(1) Diagonal | | | | 21.43 | 1.77 |
| AR(1) Rho | | | | 0.54 | 0.04 |
| UN (1,1) | | | | 12.83 | 3.16 |
| UN (2,1) | | | | 1.12 | 0.33 |
| UN (2,2) | | | | 0.23 | 0.07 |

(ASRS = Adult ADHD Self-Report Scale.)**Table S6. *Full model output of moderation analysis of reading speed***

| Fixed effects | | | | | |
| --- | --- | --- | --- | --- | --- |
|  | Est/Beta | SE | 95% CI | t | p |
| Intercept | 23.53 | 0.48 | 22.58 – 24.48 | 48.86 | < 0.001 |
| Time | -0.84 | 0.07 | -0.97 – -0.70 | -12.32 | < 0.001 |
| Group | 0.03 | 0.68 | -1.32 – 1.38 | 0.05 | 0.96 |
| DLS | -0.05 | 0.06 | -0.16 – 0.07 | -0.80 | 0.42 |
|  |  | | | | |
| Time × group | -0.04 | 0.10 | -0.23 – 0.16 | -0.36 | 0.72 |
| Time × DLS | -0.00 | 0.01 | -0.03 – 0.02 | -0.44 | 0.66 |
| Time × group × DLS | 0.03 | 0.02 | -0.00 – 0.06 | 1.86 | 0.06 |
|  | | | | | |
| No. of modules assigned | -0.28 | 0.10 | -0.49 – -0.08 | -2.70 | 0.01 |
|  | | | | | |
| Random effects | | | | | |
|  | | | | Estimate | SE |
| AR(1) Diagonal | | | | 21.60 | 1.83 |
| AR(1) Rho | | | | 0.55 | 0.04 |
| UN (1,1) | | | | 16.03 | 3.46 |
| UN (2,1) | | | | 0.97 | 0.35 |
| UN (2,2) | | | | 0.22 | 0.07 |

(DLS = *Diagnostiska Läs- och Skrivprov* (Diagnostic manual for analysis of reading and writing skills). VAS = Visual analogue scale.)

**3. Full model output from mixed-effect models without controlling for number of modules assigned**

**Table S7. Full model output of depressive symptoms**

| Fixed effects | | | | | |
| --- | --- | --- | --- | --- | --- |
|  | Est/Beta | SE | 95% CI | t | p |
| Intercept | 23.94 | 0.60 | 22.76 – 25.12 | 39.84 | < 0.001 |
| Time | -1.00 | 0.07 | -1.14 – -0.87 | -14.37 | < 0.001 |
| Time sq | 0.01 | 0.00 | 0.01 – 0.02 | 13.77 | < 0.001 |
| Group | -0.01 | 0.86 | -1.70 – 1.67 | -0.02 | 0.99 |
|  |  | | | | |
| Time × group | -0.05 | 0.10 | -0.25 – 0.15 | -0.51 | 0.61 |
| Time sq × group | 0.00 | 0.00 | 0.00 – 0.00 | 0.51 | 0.61 |
|  |  | | | | |
| Random effects | | | | | |
|  | | | | Estimate | SE |
| AR(1) Diagonal | | | | 26.94 | 1.79 |
| AR(1) Rho | | | | 0.63 | 0.03 |
| UN (1,1) | | | | 27.39 | 3.47 |
| UN (2,1) | | | | 0.00 | 0.06 |
| UN (2,2) | | | | 0.01 | 0.00 |

**Table S8. *Full model output for quality of life (EQ-5D VAS)***

| Fixed effects | | | | | |
| --- | --- | --- | --- | --- | --- |
|  | Est/Beta | SE | 95% CI | t | p |
| Intercept | 46.66 | 1.58 | 43.56 – 49.76 | 29.55 | < 0.001 |
| Time | 1.70 | 0.20 | 1.30 – 2.09 | 8.48 | < 0.001 |
| Time sq | -0.02 | 0.00 | -0.03 – .0.02 | -7.40 | < 0.001 |
| Group | -1.29 | 2.25 | -5.72 – 3.13 | -0.58 | 0.57 |
|  |  | | | | |
| Time × group | 0.07 | 0.30 | -0.52 – 0.66 | 0.22 | 0.83 |
| Time sq × group | -0.00 | 0.00 | -0.01 – 0.01 | -0.17 | 0.86 |
|  | | | | | |
| Random effects | | | | | |
|  | | | | Estimate | SE |
| Residual | | | | 185.51 | 14.46 |
| Intercept | | | | 151.16 | 21.77 |

(VAS = Visual analogue scale.)

**Table S9. *Full model output of moderation analysis of ADHD symptoms***

| Fixed effects | | | | | |
| --- | --- | --- | --- | --- | --- |
|  | Est/Beta | SE | 95% CI | t | p |
| Intercept | 21.62 | 0.56 | 20.51 – 22.72 | 38.50 | < 0.001 |
| Time | -0.84 | 0.09 | -1.02 – -0.65 | -8.81 | < 0.001 |
| Group | -0.18 | 0.65 | -1.45 – 1.10 | -0.27 | 0.79 |
| ASRS | 3.90 | 0.65 | 2.62 – 5.18 | 6.00 | < 0.001 |
|  |  | | | | |
| Time × group | 0.15 | 0.14 | -0.14 – 0.43 | 1.03 | 0.30 |
| Time × ASRS | 0.00 | 0.14 | -0.26 – 0.27 | 0.03 | 0.98 |
| Time × group × ASRS | -0.32 | 0.20 | -0.71 – 0.07 | -1.61 | 0.11 |
|  | | | | | |
| Random effects | | | | | |
|  | | | | Estimate | SE |
| AR(1) Diagonal | | | | 21.57 | 1.80 |
| AR(1) Rho | | | | 0.55 | 0.04 |
| UN (1,1) | | | | 12.78 | 3.20 |
| UN (2,1) | | | | 1.26 | 0.33 |
| UN (2,2) | | | | 0.23 | 0.07 |

(ASRS = Adult ADHD Self-Report Scale.)**Table S10. *Full model output of moderation analysis of reading speed***

| Fixed effects | | | | | |
| --- | --- | --- | --- | --- | --- |
|  | Est/Beta | SE | 95% CI | t | p |
| Intercept | 23.60 | 0.48 | 22.65 – 24.55 | 48.87 | < 0.001 |
| Time | -0.84 | 0.07 | -0.97 – -0.71 | -12.33 | < 0.001 |
| Group | 0.05 | 0.69 | -1.30 – 1.40 | 0.07 | 0.94 |
| DLS | -0.06 | 0.06 | -0.17 – 0.05 | -1.07 | 0.29 |
|  |  | | | | |
| Time × group | -0.03 | 0.10 | -0.23 – 0.16 | -0.35 | 0.73 |
| Time × DLS | 0.00 | 0.01 | -0.03 – 0.02 | -0.41 | 0.68 |
| Time × group × DLS | 0.03 | 0.02 | -0.00 – 0.06 | 1.83 | 0.07 |
|  | | | | | |
| Random effects | | | | | |
|  | | | | Estimate | SE |
| AR(1) Diagonal | | | | 21.78 | 1.87 |
| AR(1) Rho | | | | 0.55 | 0.04 |
| UN (1,1) | | | | 16.09 | 3.52 |
| UN (2,1) | | | | 1.14 | 0.34 |
| UN (2,2) | | | | 0.21 | 0.07 |

(DLS = *Diagnostiska Läs- och Skrivprov* (Diagnostic manual for analysis of reading and writing skills).)

**4. Description of iCBT treatment modules**

**Table S11. *Description of iCBT treatment modules and proportion of patients completing each module***

| Module | Description | Completers FULL (%) | Completers COND (%) |
| --- | --- | --- | --- |
| 1 | Description of the CBT model of depression and how CBT works | 100% | 100% |
| 2 | Behavioural activation – focus on creating positively reinforcing activities | 91% | 95.4% |
| 3 | Behavioural activation – focus on dealing with punishing activities | 81.4% | 85.6% |
| 4 | Cognitive restructuring – identifying negative automatic thoughts | 73.3% | 76.5% |
| 5 | Cognitive restructuring – Socratic questioning | 68.9% | 67.4% |
| 6 | Strategies for dealing with worry and anxiety | 62.2% | 59.1% |
| 7 | Strategies for dealing with sleep problems | 57.8% | 51.5% |
| 8 | Continued practice of the strategies learned so far | 51.1% | 46.2% |
| 9 | Summary of treatment and relapse prevention | 47.4% | 41.7% |
| 10 | Setting goals for the future | 36.3% | 35.6% |

(iCBT = Internet-based cognitive behavioural therapy. FULL = Full-text iCBT version. COND = Condensed iCBT version.)

**5. Observed means for each week, within-group effect sizes and between-group effect sizes without controlling for number of modules assigned**

**Table S12. *Observed means and standard deviations of MADRS-S for each measurement point***

|  | Full-text | | Condensed | | Total | |
| --- | --- | --- | --- | --- | --- | --- |
| Time | n | M (SD) | n | M (SD) | n | M (SD) |
| Screening | 135 | 25.88 (4.91) | 132 | 26.55 (5.72) | 267 | 26.21 (5.32) |
| Pre | 135 | 24.56 (5.36) | 132 | 24.36 (5.58) | 267 | 24.46 (5.46) |
| Week 1 | 125 | 22.37 (6.24) | 115 | 22.25 (6.33) | 240 | 22.31 (6.27) |
| Week 2 | 116 | 21.42 (7.00) | 113 | 20.92 (6.83) | 229 | 21.17 (6.91) |
| Week 3 | 109 | 20.51 (8.21) | 100 | 20.43 (6.86) | 209 | 20.47 (7.57) |
| Week 4 | 102 | 19.02 (7.85) | 97 | 18.87 (7.83) | 199 | 18.94 (7.82) |
| Week 5 | 93 | 18.05 (8.06) | 91 | 18.75 (7.89) | 184 | 18.40 (7.96) |
| Week 6 | 96 | 18.46 (8.25) | 88 | 17.30 (8.45) | 184 | 17.90 (8.34) |
| Week 7 | 87 | 17.48 (8.00) | 75 | 15.79 (7.61) | 162 | 16.70 (7.85) |
| Week 8 | 84 | 15.93 (8.08) | 75 | 15.23 (7.42) | 159 | 15.60 (7.76) |
| Week 9 | 84 | 15.55 (8.10) | 77 | 14.48 (7.55) | 161 | 15.04 (7.84) |
| Week 10 | 73 | 14.75 (7.79) | 68 | 14.03 (6.95) | 141 | 14.40 (7.38) |
| Post | 102 | 14.52 (8.21) | 81 | 13.77 (7.71) | 183 | 14.19 (7.98) |
| Follow-up | 84 | 14.54 (9.75) | 56 | 13.18 (8.41) | 140 | 13.99 (9.23) |

(MADRS-S = Montgomery-Åsberg Depression Rating Scale – Self-rating version.)

**Table S13. *Within-group effect sizes for the two iCBT versions compared to earlier studies using the full-text iCBT version***

|  | Pre- to post-treatment Cohen’s d (95% CI) | Pre-treatment to follow-up Cohen’s d (95% CI) |
| --- | --- | --- |
| Full-text iCBT | 1.33 (0.96 – 1.71) | 1.20 (0.83 – 1.57) |
| Condensed iCBT | 1.43 (1.05 – 1.81) | 1.33 (0.96 – 1.71) |
|  |  |  |
| Andersson et al. (13) | 1.04 (0.34 – 1.74) | 0.72 (0.05 – 1.39) |
| Vernmark et al. (14) | 1.11 | 1.54 |
| Hedman et al. (15) | 1.27 (1.14 – 1.39) | 1.28 (1.15 – 1.40) |

*Note.* 95% Effect sizes from Andersson et al were calculated based on Table 2 from that article. Confidence intervals from Vernmark et al were not reported.

**Table S14. *Between-group effect sizes for depressive symptoms and EQ-5D VAS without controlling for number of assigned modules***

|  | Endpoint | Follow up |
| --- | --- | --- |
| Depressive symptoms | -0.09 (-0.43 – 0.24) | -.07 (-.60 - .47) |
| EQ-5D VAS | 0.04 (-0.26. – 0.34) | 0.06 (-0.27. – 0.39) |

(VAS = Visual analogue scale.)

**6. Knowledge test**

**Table S15. *Items on the knowledge test – translated from Swedish***

| 1. Motivation usually appear on its own if you just wait for it. | False |
| --- | --- |
| 2. Starting with a smaller part of a task is a good way to get going. | True |
| 3. Black-and-white thinking lays the foundation for perfectionism. | True |
| 4. Labelling yourself and others helps to create a stable and predictable life. | False |
| 5. Waiting to do a task until it feels right is a good way to reduce depression since it reduces the likelihood of failure. | False |
| 6. Focusing on your bad attributes helps you stop behaving in unhelpful ways. | False |
| 7. Doing activities that increases positive emotions can make you less depressed. | True |
| 8. Everyone’s mood is improved by the same activities. | False |
| 9. Writing down your negative automatic thoughts makes you more depressed. | False |
| 10. To lessen your fear, you should avoid things you are afraid of doing. | False |
| 11. If you only do what you have to, you will not become depressed. | False |
| 12. An adult is the way he/she is and cannot learn new things. | False |
| 13. You can get energy by doing an activity. | True |
| 14. An activity can be both positive and negative at the same time. | True |
| 15. A daytime nap can make it harder to sleep at night. | True |
| 16. The important part of questioning your automatic thoughts is that so you can always think positive. | False |
| 17. Your sleep improves if you use the time you are awake at night, and cannot fall asleep, to do something fun or eat something tasty. | False |
| 18. The most important thing about setting goals is to aim as high as possible. | False |
| 19. Behavioural activation is about finding the relationship between your activities and your mood. | True |
| 20. Avoiding making other people disappointed can lead to depression. | True |

**7. Moderation sensitivity analyses**

**7.1 Sensitivity analysis of moderating effect of reading speed**

One concern is that a moderating effect of reading speed is confounded by depression severity. It could be that participants with a lower reading speed have a more severe form of depression. In that case, a moderating effect could be because of this, rather than reading speed. To test this we correlated reading speed with score of MADRS-S at pre-treatment. These variables were not significantly correlated, Pearson’s r = -0.04, p = 0.48.

Furthermore, we conducted a new moderation analysis while controlling for score of MADRS-S at pre-treatment. The three-way interaction of time, treatment group, and reading speed in this analysis was not significant, see table S16.

**7.2 Reading speed and adherence/engagement**

To further understand the effect of reading speed on treatment outcome, we conducted correlation analysis of reading speed on measures of adherence and engagement in treatment. The following measures were analysed: time spent on treatment, number of modules assigned, treatment satisfaction, result of knowledge test at post-treatment and follow-up, number of messages sent from the patient and from the therapist.

In the full sample, only the correlation between reading speed and the results of the knowledge test at post-treatment was significant, Pearson’s r = 0.25, p = < .001.

When looking at the correlation in each treatment group, there was no significant correlation in the group receiving the full-text treatment, Pearson r = 0.19, p = 0.062. However, there was a larger and significant correlation in the group receiving condensed iCBT, Pearson’s r = 0.33, p = 0.004. This indicates that higher reading speed had a positive effect on knowledge acquisition in the condensed iCBT version. No other correlations were statistically significant in either treatment group. The relationship between reading speed and acquired knowledge during treatment is of interest and future research could increase our understanding of its role in iCBT outcomes.

**Table S16. *Full model output of sensitivity analysis of moderation of reading speed on depressive symptoms***

| Fixed effects | | | | | |
| --- | --- | --- | --- | --- | --- |
|  | Est/Beta | SE | 95% CI | t | p |
| Intercept | 23.48 | 0.48 | 22.53 – 24.43 | 48.58 | < 0.001 |
| Time | -0.84 | 0.07 | -0.97 – -0.70 | -12.25 | < 0.001 |
| Group | 0.09 | 0.69 | -1.26 – 1.44 | 0.13 | 0.89 |
| DLS | -0.04 | 0.06 | -0.16 – 0.07 | -0.77 | 0.44 |
|  |  | | | | |
| Time × group | -0.04 | 0.10 | -0.23 – 0.16 | -0.38 | 0.70 |
| Time × DLS | -0.01 | 0.01 | -0.03 – 0.02 | -0.44 | 0.66 |
| Time × group × DLS | 0.03 | 0.02 | -0.00 – 0.06 | 1.86 | 0.06 |
|  | | | | | |
| No. of modules completed | -0.27 | 0.11 | -0.48 – -0.06 | -2.59 | 0.01 |
| MADRS-S pre-treatment | 0.02 | 0.04 | -0.05 – 0.08 | 0.43 | 0.67 |
| Random effects | | | | | |
|  | | | | Estimate | SE |
| AR(1) Diagonal | | | | 21.50 | 1.81 |
| AR(1) Rho | | | | 0.54 | 0.04 |
| UN (1,1) | | | | 16.18 | 3.46 |
| UN (2,1) | | | | 0.95 | 0.35 |
| UN (2,2) | | | | 0.22 | 0.07 |

(DLS = *Diagnostiska Läs- och Skrivprov* (Diagnostic manual for analysis of reading and writing skills). MADRS-S = Montgomery-Åsberg Depression Rating Scale – Self-rating version.)

**8. Sensitivity analysis of moderation of ADHD-symptoms**

The main moderation analysis of ADHD-symptoms used a dichotomous variable based on established guidelines for interpreting the ASRS-VI.I screening questionnaire (16). Since dichotomizing a variable reduces the information used in the analysis, the lack of effect could be due to this. Furthermore, the questionnaire contains questions about both inattention and hyperactivity, and these subscales could have different relationships to the treatment versions.

Therefore, we conducted three new moderation analyses to account for these possibilities. The first analysis added each item on ASRS together, with each item having a range of 0–4, meaning the total range of the scale was 0–24. We also conducted two analyses using only the questions regarding inattention or hyperactivity. For inattention, this meant the scale had a range of 0–16, and for hyperactivity 0–8. All variables were grand mean centred.

None of these sensitivity analyses showed a significant result. For the whole scale the three-way interaction was non-significant, F(1, 209.891) = 2.25, p = 0.14, and the same was true for the inattention scale, F(1, 204.496) = 1.29, p = 0.26, and hyperactivity scale, F(1, 211.598) = 1.38, p = 0.24.

**9. Difference in missing data**

A chi-square analysis was made to investigate whether the differences in missing data between the treatment groups was statistically significant. The results showed that there was a significant association between treatment group and level of missing data at both post-treatment, χ^2^ (1) = 6.234, p = 0.013, and follow-up, χ^2^ (1) = 10.48, p = 0.001. Based on the odds ratio, the odds of missing data in the condensed iCBT treatment group compared to the full-text treatment group was 2.04 times higher at post-treatment and 2.24 times higher at follow-up.

From our data there is no clear reason for this pattern. Previous research has pointed to being male, a lower education level, comorbid anxiety, and lower age as predictors of dropout (17). In our study these factors were similar between the groups. This highlights the need for our results to be replicated before drawing firm conclusions about the effect of text length in iCBT for depression. Furthermore, more research on reasons for dropout in iCBT is warranted.

**10. Non-inferiority figures**

***
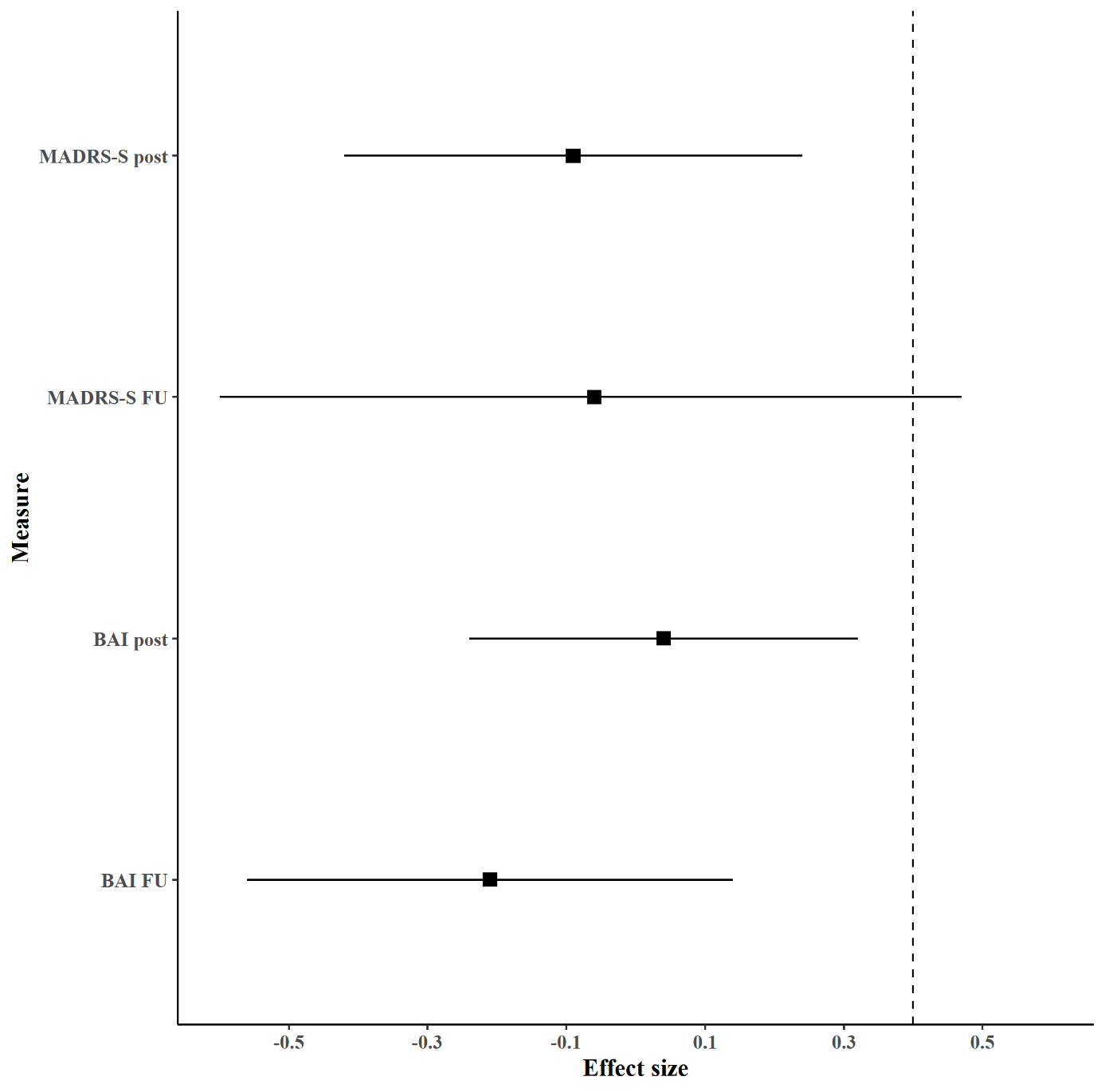
***

**Figure S1. *Between-group effect sizes and their confidence intervals of depressive and anxiety symptoms***

(MADRS-S = Montgomery Åsberg Depression Rating Scale – Self-rating version. BAI = Beck Anxiety Inventory. Post = Post-treatment measurement. FU = One-year follow-up measurement.)


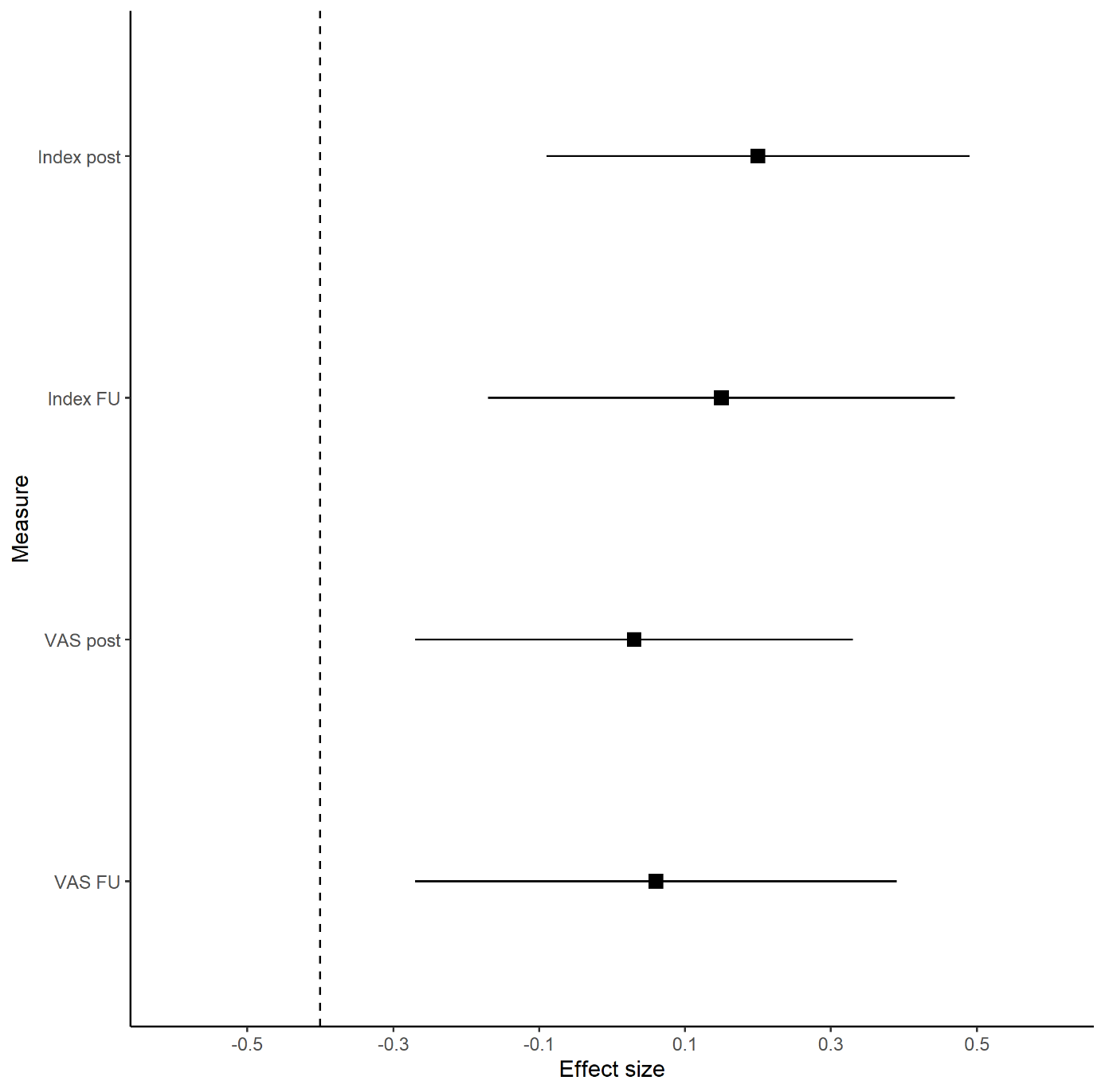


**Figure S2. *Between-group effect sizes and their confidence intervals of quality of life measures***

(Index = EQ-5D index. VAS = EQ-5D Visual Analogue Scale. Post = Post-treatment measurement. FU = One-year follow-up measurement.)

**11. Full model output of post-hoc two-way interaction analyses of moderation**

**Table S17. *Full model output of two-way interaction of ADHD symptoms in full-text iCBT version***

| Fixed effects | | | | | |
| --- | --- | --- | --- | --- | --- |
|  | Est/Beta | SE | 95% CI | t | p |
| Intercept | 21.44 | 0.63 | 20.19 – 22.69 | 33.90 | < 0.001 |
| Time | -0.84 | 0.09 | -1.02 – -0.65 | -8.95 | < 0.001 |
| ASRS | 4.12 | 0.90 | 2.35 – 5.89 | 4.60 | < 0.001 |
|  |  | | | | |
| Time × ASRS | -0.01 | 0.13 | -0.27 – 0.26 | -0.04 | 0.97 |
|  | | | | | |
| No. of modules assigned | -0.14 | 0.13 | -0.41 – 0.12 | -1.05 | 0.30 |
|  | | | | | |
| Random effects | | | | | |
|  | | | | Estimate | SE |
| AR(1) Diagonal | | | | 23.11 | 2.76 |
| AR(1) Rho | | | | 0.56 | 0.05 |
| UN (1,1) | | | | 10.19 | 4.52 |
| UN (2,1) | | | | 1.22 | 0.45 |
| UN (2,2) | | | | 0.18 | 0.09 |

(ASRS = Adult ADHD Self-Report Scale.)

**Table S18. *Full model output of two-way interaction of ADHD symptoms in condensed iCBT version***

| Fixed effects | | | | | |
| --- | --- | --- | --- | --- | --- |
|  | Est/Beta | SE | 95% CI | t | p |
| Intercept | 21.60 | 0.71 | 20.19 – 23.00 | 30.39 | < 0.001 |
| Time | -0.69 | 0.11 | -0.92 – -0.47 | -6.16 | < 0.001 |
| ASRS | 3.43 | 0.95 | 1.55 – 5.31 | 3.61 | < 0.001 |
|  |  | | | | |
| Time × ASRS | -0.30 | 0.15 | -0.60 – -0.01 | -2.03 | 0.05 |
|  | | | | | |
| No. of modules assigned | -0.33 | 0.15 | -0.62 – -0.03 | -2.21 | 0.03 |
|  | | | | | |
| Random effects | | | | | |
|  | | | | Estimate | SE |
| AR(1) Diagonal | | | | 19.25 | 2.16 |
| AR(1) Rho | | | | 0.51 | 0.06 |
| UN (1,1) | | | | 16.02 | 4.42 |
| UN (2,1) | | | | 0.91 | 0.49 |
| UN (2,2) | | | | 0.30 | 0.10 |

(ASRS = Adult ADHD Self-Report Scale.)

**Table S19. *Full model output of two-way interaction of reading speed in full-text iCBT version***

| Fixed effects | | | | | |
| --- | --- | --- | --- | --- | --- |
|  | Est/Beta | SE | 95% CI | t | p |
| Intercept | 23.52 | 0.48 | 22.57 – 24.47 | 48.88 | < 0.001 |
| Time | -0.84 | 0.07 | -0.97 – -0.71 | -12.50 | < 0.001 |
| DLS | -0.4 | 0.08 | -0.20 – 0.12 | -0.51 | 0.61 |
|  |  | | | | |
| Time × DLS | -0.1 | 0.01 | -0.03 – 0.02 | -0.45 | 0.65 |
|  | | | | | |
| No. of modules assigned | -0.20 | 0.14 | -0.48 – 0.09 | -1.37 | 0.17 |
|  | | | | | |
| Random effects | | | | | |
|  | | | | Estimate | SE |
| AR(1) Diagonal | | | | 23.41 | 2.86 |
| AR(1) Rho | | | | 0.57 | 0.05 |
| UN (1,1) | | | | 14.12 | 5.07 |
| UN (2,1) | | | | 1.22 | 0.48 |
| UN (2,2) | | | | 0.17 | 0.09 |

(DLS = *Diagnostiska Läs- och Skrivprov* (Diagnostic manual for analysis of reading and writing skills). VAS = Visual analogue scale.)

**Table S20. *Full model output of two-way interaction of reading speed in condensed iCBT version***

| Fixed effects | | | | | |
| --- | --- | --- | --- | --- | --- |
|  | Est/Beta | SE | 95% CI | t | p |
| Intercept | 23.52 | 0.50 | 22.54 – 24.51 | 47.26 | < 0.001 |
| Time | -0.87 | 0.07 | -1.02 – -0.73 | -11.89 | < 0.001 |
| DLS | -0.04 | 0.08 | -0.20 – 0.12 | -0.49 | 0.63 |
|  |  | | | | |
| Time × DLS | 0.02 | 0.01 | 0.00 – 0.05 | 2.07 | 0.04 |
|  | | | | | |
| No. of modules assigned | -0.36 | 0.16 | -0.67 – -0.05 | -2.30 | 0.02 |
|  | | | | | |
| Random effects | | | | | |
|  | | | | Estimate | SE |
| AR(1) Diagonal | | | | 19.36 | 2.22 |
| AR(1) Rho | | | | 0.51 | 0.06 |
| UN (1,1) | | | | 18.96 | 4.79 |
| UN (2,1) | | | | 0.64 | 0.51 |
| UN (2,2) | | | | 0.29 | 0.10 |

(DLS = *Diagnostiska Läs- och Skrivprov* (Diagnostic manual for analysis of reading and writing skills). VAS = Visual analogue scale.)

**Referenses**

1. Cohen J. Statistical power analysis for the behavioral sciences. 2^nd^ ed. Routledge; 1988. https://doi.org/10.4324/9780203771587

2. Hedman E, Andersson G, Ljotsson B, Andersson E, Ruck C, Mortberg E, et al. Internet-based cognitive behavior therapy vs. cognitive behavioral group therapy for social anxiety disorder: A randomized controlled non-inferiority trial. PLoS ONE [Electronic Resource]. 2011;6(3). http://dx.doi.org/10.1371/journal.pone.0018001

3. Elashoff JD. nQuery Advisor Version 4.0 User's Guide. Boston: Statistical Solutions; 2000.

4. Hesser H. Modeling individual differences in randomized experiments using growth models: Recommendations for design, statistical analysis and reporting of results of internet interventions. Internet interventions. 2015;2(2):110-20. https://doi.org/10.1016/j.invent.2015.02.003

5. Biesanz JC, Deeb-Sossa N, Papadakis AA, Bollen KA, Curran PJ. The role of coding time in estimating and interpreting growth curve models. Psychol Methods. 2004;9(1):30. https://psycnet.apa.org/doi/10.1037/1082-989X.9.1.30

6. Luke SG. Evaluating significance in linear mixed-effects models in R. Behav Res Methods. 2017;49(4):1494-502. https://doi.org/10.3758/s13428-016-0809-y

7. Karin E, Crane MF, Dear BF, Nielssen O, Heller GZ, Kayrouz R, et al. Predictors, outcomes, and statistical solutions of missing cases in web-based psychotherapy: methodological replication and elaboration study. JMIR Mental Health. 2021;8(2):e22700. https://doi.org/10.2196/22700

8. Karin E, Dear BF, Heller GZ, Crane MF, Titov N. “Wish you were here”: examining characteristics, outcomes, and statistical solutions for missing cases in web-based psychotherapeutic trials. JMIR Mental Health. 2018;5(2):e8363. https://doi.org/10.2196/mental.8363

9. Feingold A. Effect of parameterization on statistical power and effect size estimation in latent growth modeling. Structural Equation Modeling: A Multidisciplinary Journal. 2021;28(4):609-21. https://doi.org/10.1080/10705511.2021.1878895

10. IBM SPSS Statistics for Windows. 27.0 ed. Armonk, NY: IBM Corp; 2020

11. R Core Team. R: A language and environment for statistical computing [Internet]. Version 4.0. Vienna, Austria; 2019. Available from: https://www.R-project.org.

12. Wickham H. ggplot2: Elegant Graphics for Data Analysis: Springer-Verlag New York; 2016.

13. Andersson G, Bergström J, Holländare F, Carlbring P, Kaldo V, Ekselius L. Internet-based self-help for depression: randomised controlled trial. The British Journal of Psychiatry. 2005;187(5):456-61. https://doi.org/10.1192/bjp.187.5.456

14. Vernmark K, Lenndin J, Bjärehed J, Carlsson M, Karlsson J, Öberg J, et al. Internet administered guided self-help versus individualized e-mail therapy: A randomized trial of two versions of CBT for major depression. Behav Res Ther. 2010;48(5):368-76. https://doi.org/10.1016/j.brat.2010.01.005

15. Hedman E, Ljótsson B, Kaldo V, Hesser H, El Alaoui S, Kraepelien M, et al. Effectiveness of Internet-based cognitive behaviour therapy for depression in routine psychiatric care. J Affect Disord. 2014;155:49-58. https://doi.org/10.1016/j.jad.2013.10.023

16. Kessler RC, Adler L, Ames M, Demler O, Faraone S, Hiripi E, et al. The World Health Organization Adult ADHD Self-Report Scale (ASRS): a short screening scale for use in the general population. Psychol Med. 2005;35(2):245. https://doi.org/10.1017/S0033291704002892

17. Karyotaki E, Kleiboer A, Smit F, Turner DT, Pastor AM, Andersson G, et al. Predictors of treatment dropout in self-guided web-based interventions for depression: an ‘individual patient data’ meta-analysis. Psychol Med. 2015;45(13):2717-26. https://doi.org/10.1017/S0033291715000665
